# Enteroscopic Diagnosis and Management of Small Bowel Diverticular Hemorrhage: A Multicenter Report from the Taiwan Association for the Study of Small Intestinal Diseases

**DOI:** 10.1155/2015/564536

**Published:** 2015-08-13

**Authors:** Yang-Yuan Chen, Cheng-Tang Chiu, Chen-Ming Hsu, Tsung-Hsing Chen, Yi-Chun Chiu, Yen-Chang Chu, Chen-Wang Chang, Hsiu-Po Wang, Deng-Chyang Wu, Tien-Yu Huang, Hsu-Heng Yen

**Affiliations:** ^1^Division of Gastroenterology, Department of Internal Medicine, China Medical University Hospital, Taichung 40447, Taiwan; ^2^Endoscopy Center, Department of Gastroenterology, Changhua Christian Hospital, Changhua 50000, Taiwan; ^3^Department of Gastroenterology and Hepatology, Linkou Chang Gung Memorial Hospital, Chang Gung University College of Medicine, Taoyuan 33344, Taiwan; ^4^Division of Hepato-Gastroenterology, Department of Internal Medicine, Kaohsiung Chang Gung Memorial Hospital and Chang Gung University College of Medicine, Kaohsiung 83301, Taiwan; ^5^Department of Gastroenterology, Buddhist Taichung Tzu Chi Hospital, Taichung 42700, Taiwan; ^6^Division of Gastroenterology, Department of Internal Medicine, Mackay Memorial Hospital, Taipei 10449, Taiwan; ^7^Department of Internal Medicine, National Taiwan University Hospital, Taipei 10002, Taiwan; ^8^Department of Internal Medicine, Kaohsiung Medical University Hospital, Kaohsiung 80700, Taiwan; ^9^Division of Gastroenterology, Tri-Service General Hospital, Taipei 11400, Taiwan

## Abstract

Small bowel diverticulum is a rare cause of gastrointestinal bleeding. The diagnosis and treatment of small bowel diverticular hemorrhage is clinically challenging before the development of deep enteroscopy. In this multicenter study from the Taiwan Association for the Study of Small Intestinal Diseases (TASSID), 608 patients underwent deep enteroscopy for obscure gastrointestinal bleeding during January 2004 and April 2010 from eight medical centers in Taiwan. Small bowel diverticular hemorrhage account for 7.89% of obscure gastrointestinal bleeding in this study. Most of the patients received endoscopic therapy with an initial hemostasis rate of 85.71% and rebleeding rate of 20%. In this large case series investigating the enteroscopic management of small intestinal diverticular hemorrhage, we found that, as to patients with peptic ulcer hemorrhage, most of these patients can be successfully managed by endoscopic therapy before surgery in the era of deep enteroscopy.

## 1. Introduction

Small bowel diverticula are a rare cause of gastrointestinal bleeding [1–4]; they usually occur in the duodenum (followed by jejunum and ileum) and most patients are asymptomatic [3, 5]. Although rare, most bleeding complications from duodenal diverticula can be endoscopically managed [6, 7] within the confines of conventional or push endoscopy. In contrast, small bowel diverticula located distal to the proximal duodenum are less approachable by conventional endoscopic techniques. Complications arising from these jejunoileal diverticula, particularly hemorrhage, are clinically challenging in both their diagnosis and management [1, 2, 8, 9]. Only 49 cases of bleeding complications associated with jejunoileal diverticula were reported in 1987 [10, 11], and this number increased to 60 in 1992 [12]. With increased awareness of this rare disorder and improved diagnostic tools such as capsule endoscopy and deep enteroscopy [13–16], the number of cases reported in the literature has increased.

In the past, radiological examination followed by surgical resection of the involved bowel was the only recommended approach to treat this rare phenomenon [17]. Improvements in endoscopic technology during the 21st century have changed the approach towards diagnosis and management of various small bowel diseases [5, 18, 19]. However, studies on the endoscopic diagnosis and management of small bowel diverticular bleeding have mainly been limited to case reports [14, 15, 20] from Asia. Two case series in Taiwan [5, 16] reported the utility of double-balloon enteroscopy in the diagnosis and management of this rare disorder. To further clarify the role of deep enteroscopy in the management of small bowel diverticular hemorrhage, a committee from the Taiwan association for the study of small intestinal diseases (TASSID) decided to conduct a retrospective study to investigate the clinical features, management, and treatment outcome of small bowel diverticular hemorrhage in Taiwan.

## 2. Patients and Methods

A standard questionnaire was developed in 2010 by Dr. Yang-Yuan Chen to collect clinical information on patients diagnosed with small bowel diverticular hemorrhage. All member centers of the Taiwan association for the study of small intestinal diseases (TASSID) were invited to report their experience of enteroscopic management of this disorder between January 2004 and April 2010. Eight centers in which deep enteroscopy (single-balloon, *n* = 2; double-balloon, *n* = 6) had been performed and used for management of small bowel diverticular hemorrhage were enrolled in this multicenter study. Diagnosis of this disorder was made according to the following criteria: (a) the presence of a small bowel diverticulum discerned by radiology, endoscopy, or surgical findings; (b) the absence of bleeding from the esophagus, stomach, duodenum, or colon after endoscopic evaluation; and (c) evidence of bleeding lesions associated with the diverticulum determined by endoscopic, radiological, or surgical evaluation. Patients with Meckel's diverticulum were excluded.

## 3. Results

### 3.1. Clinical Features

Forty-eight (21 male, 27 female) of 608 patients (7.89%) who underwent enteroscopy in eight participating centers and were diagnosed with small bowel diverticular hemorrhage (range, 1–18 patients per center) were analyzed in the study. The baseline characteristics of the patients are summarized in Table 1. Their mean age was 71.42 ± 12.35 years and their symptoms included bloody stools (29.2%) and tarry stools (79.2%). The median duration of stay in hospital was 14.88 days (range, 0–80) and the median blood transfusion volume was four units (range, 0–26).

The diverticula were mainly located in the jejunum, followed by the distal duodenum and ileum. A single diverticulum was detected in 56.16% of patients, and the remainder had multiple diverticula. Preenteroscopic evaluations included computerized tomography (*n* = 17), small bowel follow-through (*n* = 21), and capsule endoscopy (*n* = 9).

### 3.2. Enteroscopic Features and Treatment Outcome

**Figure 1:**
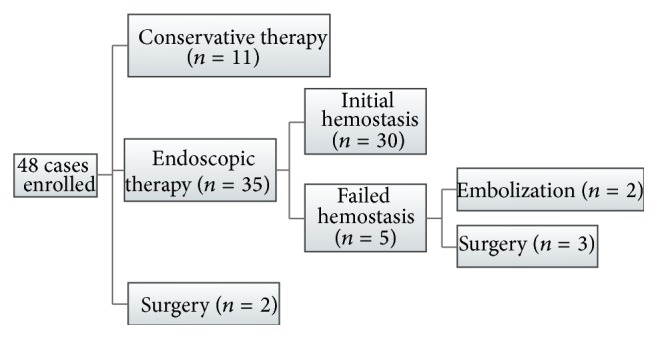
Flow chart of patient management and outcome.

During enteroscopic examination, 25% (12/48) of patients were found to have ulcers without bleeding associated with the diverticulum and the remaining (75%) patients were found to have active bleeding or stigmata of recent hemorrhage, that is, blood clots or protruding vessels. The patient management and outcome were summarized in Figure 1. Two patients were referred for surgery after diagnosis and 11 patients received conservative medical therapy, including parenteral nutrition and/or tranexamic acid therapy. Most of the patients (35/48) received endoscopic therapy as a first-line treatment to control bleeding. A single endoscopic procedure, such as argon plasma coagulation (*n* = −9), hemoclip (*n* = 7), or injection (*n* = 8), was performed in 68.57% (24/35) of patients and the remainder (11/35) received combined therapy. Initial hemostasis was achieved in 85.7% (30/35) and recurrent bleeding was observed in 20% (6/30) of these patients; recurrent bleeding occurred in 12.5% (6/48) during the 1-month follow-up period; and the overall bleeding-related mortality rate was 8.33% (4/48), including uncontrolled recurrent bleeding (*n* = 2), acute renal failure (*n* = 1), and multiple organ failure after angiography embolization (*n* = 1).

## 4. Discussion

Up to 70% of patients with small intestinal diverticular disease are asymptomatic [17]. Acute complications, such as bleeding, perforation, obstruction, or diverticulitis, can occur with colonic diverticula [4, 17]. Bleeding complications from small intestinal diverticula are rare and affect only 5%–33% of cases [3]. Despite their rare occurrence, overall mortality is high (14%–80%) [3, 4, 17] because of difficulties in their diagnosis and management. In the past, surgical intervention with resection of the involved bowel has been the recommended treatment of choice [3, 4, 17]. The current study is important because it is the first report on the role of enteroscopy in the diagnosis and management of this rare disorder based on a nationwide survey.

Since the introduction of double-balloon enteroscopy, diagnosis and management of various small bowel diseases have changed [21]. One of the most important functions of enteroscopy is to investigate obscure gastrointestinal bleeding [13]. While angiodysplasia or ulcers are the most common findings during this procedure [22–24], small bowel diverticular hemorrhage was found in up to 20% of patients examined for obscure gastrointestinal bleeding in the Chinese populations [5, 16]. It is unclear whether there is an ethnic predilection of the Chinese population to this disorder, as observed for duodenal diverticular hemorrhage [6]. In this nationwide survey, we found that small bowel diverticulosis accounted for 7.89% of cases of obscure gastrointestinal bleeding in Taiwan, which is similar to the results of a recent meta-analysis of the diagnostic yield of double-balloon enteroscopy [24]. A higher incidence of diverticular hemorrhage is found in eastern than in western countries (6.8% versus 1.2%) [24]. Our patients had a mean age of 71.42 years with female predominance, which suggested that small bowel diverticular hemorrhage could be a cause of obscure gastrointestinal bleeding, especially in elderly Asian patients [3, 4, 17].

The mechanisms by which small bowel diverticular hemorrhage occurs are thought to be ulceration from diverticulitis or trauma to the mesenteric vessels [3]. Angiodysplasia, tumors, or parasitic infection [3, 25] has also been reported to be associated with diverticular hemorrhage. In this series, both ulcers and Dieulafoy-like lesions were most commonly associated with bleeding, which suggested that standard endoscopic hemostatic techniques can also be applied in this setting [15, 20, 26]. Although there was no standard or consensus approach to the management of this disorder, endoscopic hemostasis was achieved in 85.7% of our patients. Before the introduction of deep enteroscopy, the management of small bowel diverticular hemorrhage has mainly been through surgical resection [1, 3, 25] and the reported mortality rate following medical treatment was higher (80%) than that after surgical treatment (14%) [17]. In this study, we also found a lower mortality rate (8.33%) than that reported previously [17]. Endoscopic therapy has revolutionized the management of peptic ulcer bleeding [16], and, similarly, the use of deep enteroscopy allows endoscopists to manage small bowel diverticular hemorrhage less invasively.

Although this nonsurgical approach controls hemorrhage, a significant number of patients (20%) reported recurrent bleeding during the follow-up period. It remains unclear whether these patients should receive surgery after initial medical management. This study is limited because only patients diagnosed by enteroscopy were included, and we have no data of patients with this disorder who did not undergo enteroscopic evaluation. Thus, a comparison between the outcomes of surgical and endoscopic therapy for this disorder is not possible. In addition, this retrospective study was limited to a follow-up period of 1 month. Although a 1-month follow-up period is a generally accepted observation time for the evaluation of patients with recurrent bleeding after peptic ulcer hemorrhage, no acceptable observation time to evaluate patients with small bowel diverticular hemorrhage has been defined. The 1-month rate of recurrent bleeding may be an underestimation of the actual rate of recurrence for this disorder. Because most of these are elderly patients, for whom surgery carries a significant risk, surgery could be reserved for patients who have uncontrolled or recurrent hemorrhage.

## 5. Conclusions

To our knowledge, this is the largest case series that has investigated the enteroscopic management of small intestinal diverticular hemorrhage. We found that, similar to patients with peptic ulcer, most of these patients can be managed by endoscopic therapy rather than surgery. Mortality remained substantial but was significantly decreased compared with that in previous studies. Advances in enteroscopic techniques offer an alternative means to diagnose and manage this rare disorder. However, a long-term follow-up is required to determine the efficacy of its endoscopic management and the retrospective nature of the study limits the generalization of these results.

## Figures and Tables

**Table 1:** Demographic data and clinical features of patients with small bowel diverticular hemorrhage (*n* = 48).

Clinical variables of patients	Data
Gender (male/female)	21/27
Age (years, mean ± SD)	71.42 ± 12.35
Hospital stay duration (days, median range)	14.88 (0–80 days)
Blood transfusion (units, median range)	4 (0–26 U)
Symptoms	
Bloody stool, *n* (%)	14 (29.2%)
Tarry stool, *n* (%)	38 (79.2%)
Number and location of the diverticula	
Distal duodenum (*n*)	6
Jejunum (*n*)	45
Ileum (*n*)	1
Single/multiple	26/22
Preendoscopic evaluations	
CT scan (*n*)	17
Small bowel follow-through (*n*)	21
Capsule endoscopy (*n*)	**9**
Endoscopic findings	
Active bleeding/blood clots/ protruding vessel (*n*)	36 (75%)
Ulcers (*n*)	12 (25%)
Bleeding-related mortality, *n* (%)	4 (8.3%)
Recurrent bleeding after hemostasis, *n* (%)	6 (20%)

## Take-Home Message

1. Small bowel diverticular hemorrhage is not an uncommon cause of obscure gastrointestinal bleeding after deep enteroscopy.
2. This nationwide survey from Taiwan revealed that management and clinical outcome of small bowel diverticula are similar to those of peptic ulcers.
3. A long-term follow-up is required to determine the efficacy of endoscopic management of this disorder.
